# Effects of scleral collagen crosslinking with different carbohydrate on chemical bond and ultrastructure of rabbit sclera: Future treatment for myopia progression

**DOI:** 10.1371/journal.pone.0216425

**Published:** 2019-05-13

**Authors:** Tae Gi Kim, Wansun Kim, Samjin Choi, Kyung Hyun Jin

**Affiliations:** 1 Department of Ophthalmology, Kyung Hee University Hospital at Gangdong, Kyung Hee University, Seoul, Korea; 2 Department of Biomedical Engineering, College of Medicine, Kyung Hee University, Seoul, Korea; 3 Department of Ophthalmology, Kyung Hee University Medical Center, Kyung Hee University, Seoul, Korea; LAAS-CNRS, FRANCE

## Abstract

**Background:**

Myopia is the most common ocular disorder and is mainly caused by axial elongation of the sclera. If the stiffness of sclera increased, it can inhibit myopia progression. The aim of this study is to compare the effect of the collagen crosslinking with different types and concentrations of carbohydrates on chemical bond and ultrastructural change of rabbit sclera.

**Methods:**

Nine New Zealand white rabbits were treated with five, sequential sub-Tenon injections of 0.15 mL solutions of ribose, sucrose, and glycogen of 0.1, 0.2 and 0.4 M concentration at the right eye over 14 days. Ten weeks after the last injection, the rabbits were sacrificed and chemical bond and ultrastructural changes were compared with those of the untreated left sclera using Raman spectroscopy, atomic force microscopy (AFM), and histology.

**Results:**

Raman spectroscopy of the control and cross-linked rabbit sclera tissue revealed different types of collagen interactions. Raman shift of 919 cm^-1^ (C-C stretching and vibration of the proline ring in collagen) was the highest in ribose, followed by sucrose and glycogen. Total energy intensity was also highest in ribose, followed by sucrose and glycogen, and showed a tendency to increase at higher concentrations. AFM revealed interlocking arrangements of collagen fibrils. The collagen fibril diameter was 105.6 ± 21.2 nm, 109.4 ± 28.8 nm, 113.1 ± 30.8 nm and 137.6 ± 25.3 nm for control group, 0.4 M glycogen, sucrose, and ribose, respectively. Histology indicated increased density of the collagen bundle and no increase in inflammatory cell recruitment compared to control at high concentrations of ribose.

**Conclusions:**

Scleral crosslinking using glycation increased the scleral biomechanical rigidity and these results were particularly pronounced in ribose. Scleral crosslinking using glycation may be a promising method for inhibiting high myopia progression.

## Introduction

Myopia is the most common ocular disorder in the world. It occurs in 85–90% of young adults in some industrialized Asian countries, such as Singapore and Taiwan, and in 25–50% of adults in the West [1]. Myopia typically occurs approximately at 8 years of age and progresses through 15–16 years of age [2,3]. High myopia more than -6 Diopter causes various complications and increases the risk of complications, such as glaucoma, retinal tear, retinal detachment, staphyloma, and myopic choroidal neovascularization [4]. Especially high myopia with posterior staphyloma has worse visual outcome than pathologic myopia without posterior staphyloma. Posterior staphyloma is known to affect the functional and anatomical properties of eyeballs and cause chorioretinal atrophy and myopic choroidal neovascularization [5].

Myopia is caused by axial elongation of the sclera. Although it is unclear if scleral weakening is a consequence or cause of myopia progression, it is known that the axial elongation is associated with hyperopic defocus of the peripheral retina along with ciliary-choroidal mechanical tension created by the crystalline lens or ciliary body during accommodation [6,7]. Several studies have reported potential therapeutic interventions to prevent myopic progression, including bifocal or multifocal spectacles, gas permeable contact lenses, topical pharmaceutical agents, orthokeratology contact lenses, and soft bifocal contact lenses [8–13].

Methods of scleral reinforcement, which directly increases the stiffness of the sclera, have also been developed and include microsurgical scleroplasty [14] and scleral collagen crosslinking induced by the photosensitizer riboflavin and ultraviolet-A (UVA) irradiation at 370 nm [15–20]. Since the biomechanically weakened sclera is one important feature of the pathologic changes of severe myopia, increasing the stiffness of the sclera represents a direct treatment of myopia progression. However, the surgical method is invasive and the process of recovery is slow. Moreover, crosslinking using UVA and riboflavin requires surgical exposure for UVA irradiation. The retina is also exposed to cytotoxic risk based upon the degree of penetration of UV light through the sclera. Indeed, previous studies have reported considerable loss in the photoreceptors and the retinal pigment epithelium using UVA and riboflavin for collagen crosslinking in rabbit sclera with 4.2 mW / cm^2^ UVA at 370 nm for 30 min [17]. Later, Wollensak et al. reported that there were no side effects on the retina or retinal pigment epithelium when a reduced energy of 3.0 mW / cm^2^ was used for 30 minutes [18]. In addition, Dotan et al reported inhibition of axial elongation in a rabbit model, and they did not observe any histological difference between untreated and treated retinal samples [20]. However, there is currently no established protocol for riboflavin-UVA crosslinking. Furthermore, it still remains a challenge to protect the optic nerve and conjunctiva from the scattered UVA light and prevent scleral drying and thinning upon exposure of the sclera during the procedure. Therefore, scleral collagen crosslinking using UVA is still requires further refinement in a pre-clinical myopia animal model [17–20].

An alternative approach is non-enzymatic glycation using a sugar molecule, such as ribose or glucose, without the controlling action of an enzyme [21]. During this process, the carbonyl group of the sugar moiety reacts with the amino group of the protein to form irreversible products (i.e., advanced glycation end-products (AGEs)) [22–24]. Carbohydrate-based collagen crosslinking is advantageous because it requires a less invasive application procedure, does not use UVA, and reduces scleral toxicity since it does not require UV exposure. In addition, carbohydrate is a natural product of metabolism and therefore poses a reduced risk of cytotoxicity. Reddy reported that the stiffness of tissue increases following non-enzymatic glycation of the rabbit Achilles tendon [25]. In a previous in vitro study, researchers found that the thickness of collagen fibrils and the rigidity of the human scleral tissue both increased following immersion in a microfluidic-based chamber containing D-ribose, which facilitated collagen crosslinking via glycation [26]. However, to our knowledge, there has been no in vivo study of scleral collagen crosslinking using a monosaccharide, disaccharide, and polysaccharide in scleral tissue. Furthermore, no study has yet investigated which of these carbohydrates is more effective for scleral crosslinking.

The purpose of this study was to analyze the chemical bond and ultrastructural change of collagen crosslinking with different types and concentrations of carbohydrates on rabbit sclera using Raman spectrometry, atomic force microscopy (AFM), and histology.

## Materials and methods

### Materials

In this study, monosaccharide ribose, disaccharide sucrose, and polysaccharide glycogen were used. The carbohydrates D-ribose (C_5_H_10_O_5,_ >99%), D-sucrose (C_12_H_22_O_11,_ >99%) and glycogen for glycation were obtained from Sigma–Aldrich (St. Louis, MO) and were prepared at concentrations of 0.1, 0.2, and 0.4 M. Ribose is a pentose monosaccharide, sucrose is a disaccharide combination of the monosaccharides glucose and fructose, and glycogen is a multi-branched polysaccharide of glucose.

### Animals

Nine male New Zealand white rabbits (3.5 kg) were treated. The crosslinked right eyes were used for biomechanical examinations and the contralateral left eyes were used as controls. This animal care and use protocol was in compliance with the ARVO Statement for the Use of Animals in Ophthalmic and Vision Research and approved by the Institutional Animal Care and Use Committee at Kyung Hee University Medical Center (KHMC-IACUC-2014-17).

### Treatment procedure

For local anesthesia, proparacaine eye drops were used. A 27 G needle was used to perform 0.15 mL sub-Tenon injections of ribose, sucrose, or glycogen solution at a depth of 3.0 mm behind the limbus in the superonasal quadrant. Sub-Tenon injections were administered 5 times over 14 days with 2 to 3 days between injections. The animals were sacrificed 10 weeks after the last injection using an overdose of intravenous sodium phenobarbital (250 mg/1.2 ml). Intramuscular 1.5 mL Zoletil (15 mg/kg) and 0.5 mL xylazine hydrochloride (5 mg/kg) were injected and enucleation was performed. Ten tissue samples obtain at the subTenon injection site were analyzed from each eye.

### Biomechanical analysis: Raman spectroscopy

The SENTERRA confocal Raman system (Bruker Optics, Billerica, MA) equipped with a 100-mW, 785-nm diode laser source with 3–5 cm^-1^ resolution was used to acquire the Raman spectra. A M Plan 100 X air objective (NA 0.9; Olympus, Tokyo, Japan) was used to focus the laser (focal spot size <1 μm) onto each fixed sample and two 30-s acquisitions were conducted. Raman spectra were analyzed using OPUS software (Bruker Optics) centered at 1200 cm^-1^ with a range of 417–1782 cm^-1^. Data were analyzed using MATLAB computing software (MathWorks, Natick, MA). All Raman spectra were baseline-corrected using a concave rubberband algorithm, which performed 10 iterations on 64 points for preliminary evaluation and peak assignments. The resulting Raman spectrum was normalized using the maximum intensity peaks. The experimental setup was designed to maximize signal intensity and minimize background noise. For quantitative analysis, the multivariate statistical techniques of principal component analysis (PCA) was performed.

### Structural analysis: AFM analysis

All AFM tapping-mode topographical images were obtained using a NANOS N8 NEOS (Bruker, Herzogenrath, Germany) equipped with a 42.5 X 42.5 X 4 μm^3^ XYZscanner and two Zeiss optical microscopes (Epiplan 200X/500X; Zena, Germany). The surface of the fixed sclera tissues mounted on a 50-nm gold-coated substrate was scanned in air with a size of 10 X 10 μm^2^ or 5 X 5 μm^2^ at a scan speed of 0.6 lines/s. AFM tapping-mode imaging was performed in 35% relative humidity at room temperature using a silicon cantilever with an integral pyramidal shaped tip (SICONG, Santa Clara, CA). The nominal tip radius and height were <10 nm and 12–16 μm, respectively.

### Histologic examination

After fixation with 4% glutaraldehyde, the specimens were embedded in paraffin. They were then sectioned on the sagittal plane and stained with Masson's trichrome to investigate the collagen fibrils.

## Results

### Raman spectra of rabbit sclera

**Fig 1:**
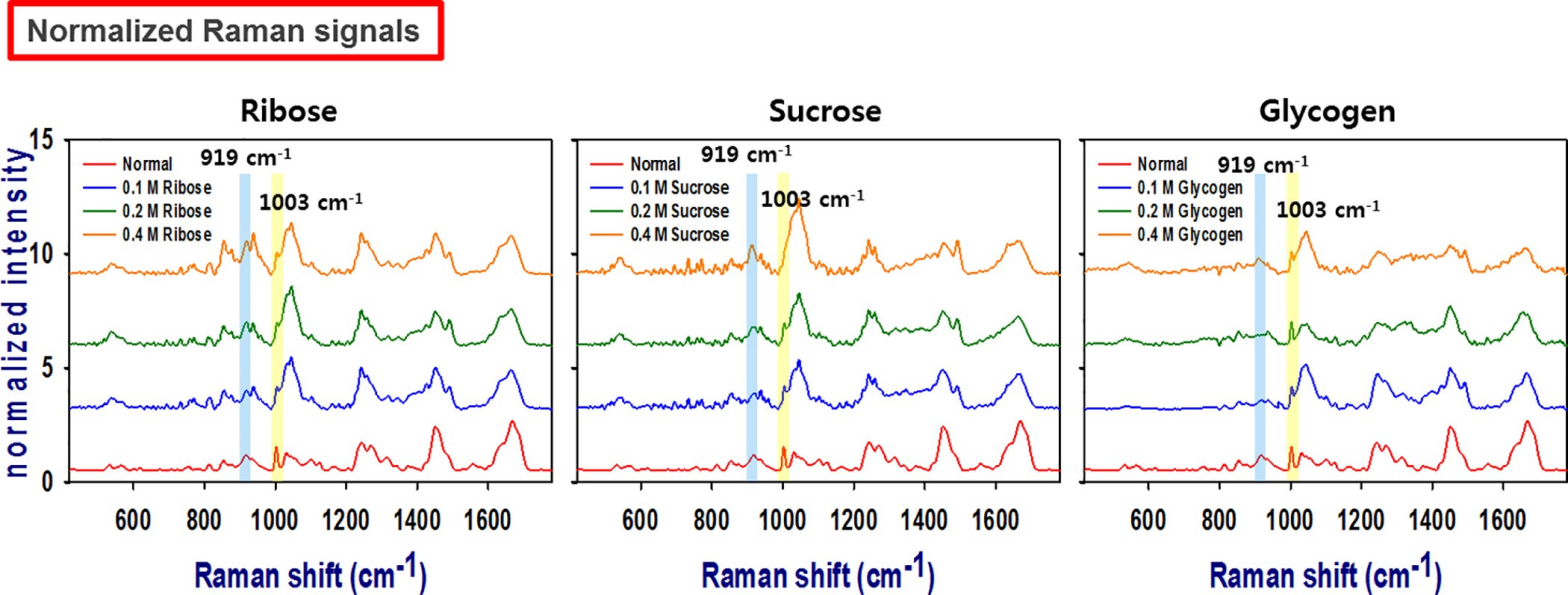
Representative Raman spectral signals of scleral tissues following treatment with 0.1, 0.2 and 0.4 M solutions of each carbohydrate. All Raman spectra were normalized at an intensity of 1003 cm^-1^ (C-C aromatic ring: phenylalanine). Glycation with carbohydrate led to a Raman shift and strong intensity at 919 cm^-1^ (C-C stretching vibration of the proline ring in collagen).

The Raman spectra of rabbit scleral tissues were shown in Fig 1. Each Raman peak showed distinct vibration characteristics of the rabbit scleral tissues. All groups showed intense peaks at 532 cm^-1^ (S─S stretching), 853 cm^-1^ (tyrosine ring breath), 919 cm^-1^ (C─C stretching of a proline ring), 1003 cm^-1^ (phenylalanine symmetric ring breath), 1031 cm^-1^ (phenylalanine), 1242 cm^-1^ (amide III b-sheet), 1448 cm^-1^ (C─H deformation in DNA/RNA, proteins, lipids, and carbohydrates), and 1669 cm^-1^ (C = O stretching of amide I). All groups showed a similar Raman peak pattern regardless of the type and concentration of carbohydrate.

### Total Raman energy in various carbohydrates

**Fig 2:**
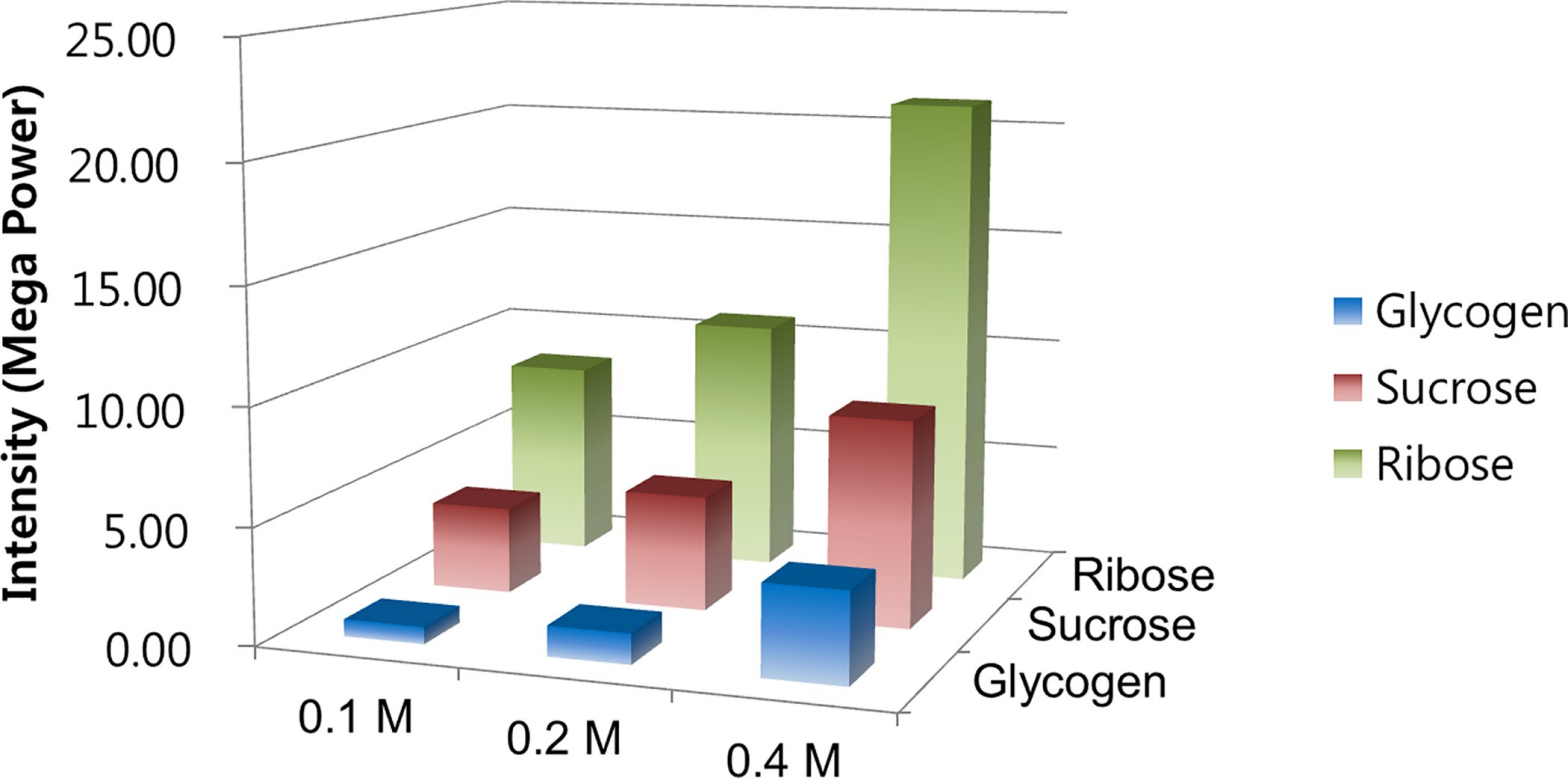
Total Raman energy of varying carbohydrates at different concentration. At each concentration, the intensity of total Raman energy was greater in the order of ribose, sucrose, and glycogen. For each carbohydrate, a concentration resulted in greater intensity of scleral collagen crosslinking.

Total energy intensities were greatest in ribose, less in sucrose, and least in glycogen when compared at the same concentrations (Fig 2). Moreover, the intensity differences between carbohydrates was greater at the concentration of 0.4 M (i.e., the disparity in total energy intensity between ribose and the other carbohydrates was greatest at higher concentrations). A higher concentration resulted in a greater total energy intensity for each carbohydrate. Moreover, the intensity difference across each concentration was greatest in the ribose group.

### Principle component analysis

**Fig 3:**
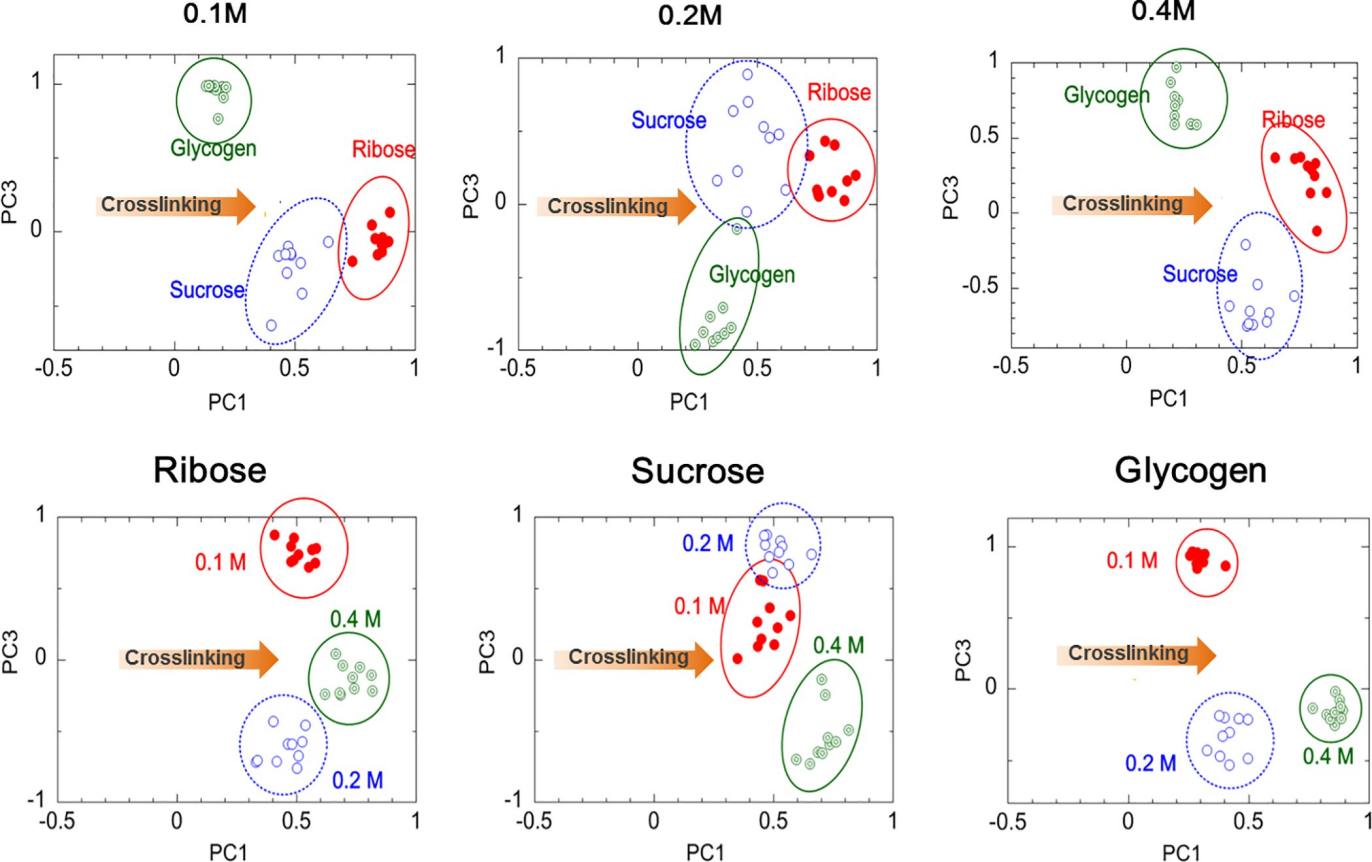
Principle component analysis of Raman spectra. PC1−PC3 used to classify the three carbohydrate molecules at three different concentrations.

PCA was utilized to identify key information in the spectra. In PCA, several peaks in the principal component (PC) loading spectra were identified as major contributors to the PC scores. Three PC loading profiles (PC1, PC2, and PC3) were extracted from ribose-, sucrose- and glycogen-treated sclera. Fig 3 shows the representative loading plots of two PC profiles. The scatter plot using PC1 and PC3 exhibited the best discrimination. The PCA algorithm demonstrated that Raman spectra were grouped according to type and concentration of carbohydrates. For each concentration, the PCA algorithm showed that the groups formed in the order of ribose, sucrose, and then glycogen along the PC1 axis (Fig 3A, 3B and 3C). However, the loading profiles of 0.2 M of sucrose or glucose were distributed along a wide region. For each carbohydrate, the PCA algorithms were grouped by carbohydrate concentration in the order of 0.4, 0.2 and 0.1 M. For each carbohydrate, the PCA algorithm for 0.4 M exhibited more clearly divided groups than did the algorithms for the remaining concentrations, whereas, between 0.1 M and 0.2 M, the PCA algorithm was distributed across a wide region.

### Structural changes

**Fig 4:**
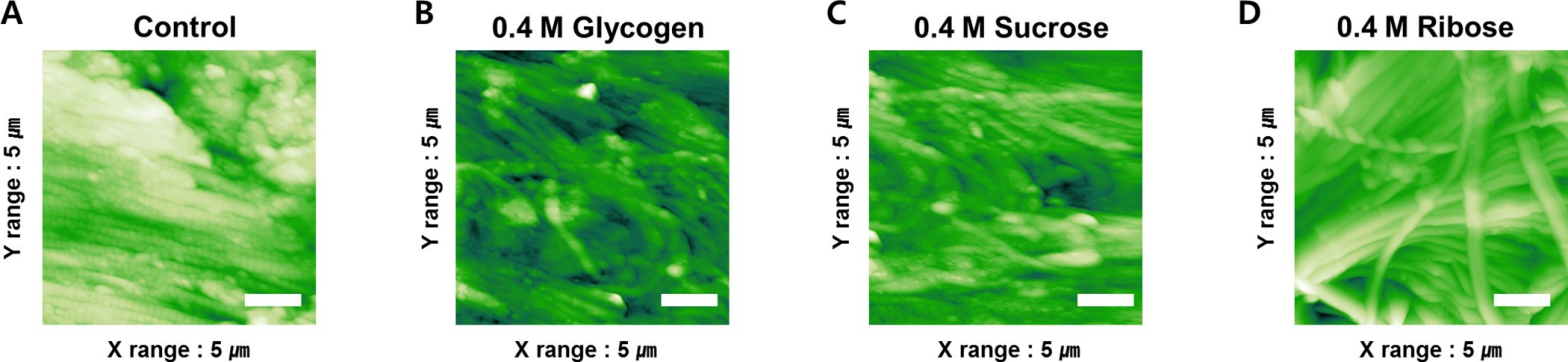
Nanostructural changes in the surface of the scleral tissues based on exposure to glycogen, sucrose, and ribose (Atomic force microscopy (AFM) tapping-mode two dimensional topographical images). Scale bar = 1 μm.

AFM tapping-mode topographical images of the rabbit scleral surfaces before and after glycation were shown in Fig 4. The AFM tapping-mode two dimensional topographical images of the control group show that typical normal scleral surfaces have a regular parallel arrangement of collagen fibrils (Fig 4A). The scleral tissues exposed to 0.4 M glycogen and 0.4 M sucrose did not show a significant difference in collagen fibril diameter compared to that of the control group; however, an irregular arrangement of collagen fibrils was noted (Fig 4B and 4C). Scleral tissues treated with a 0.4 M ribose solution showed a clear irregular parallel arrangement of the collagen fibrils with tangled fibrils (Fig 4D).

**Fig 5:**
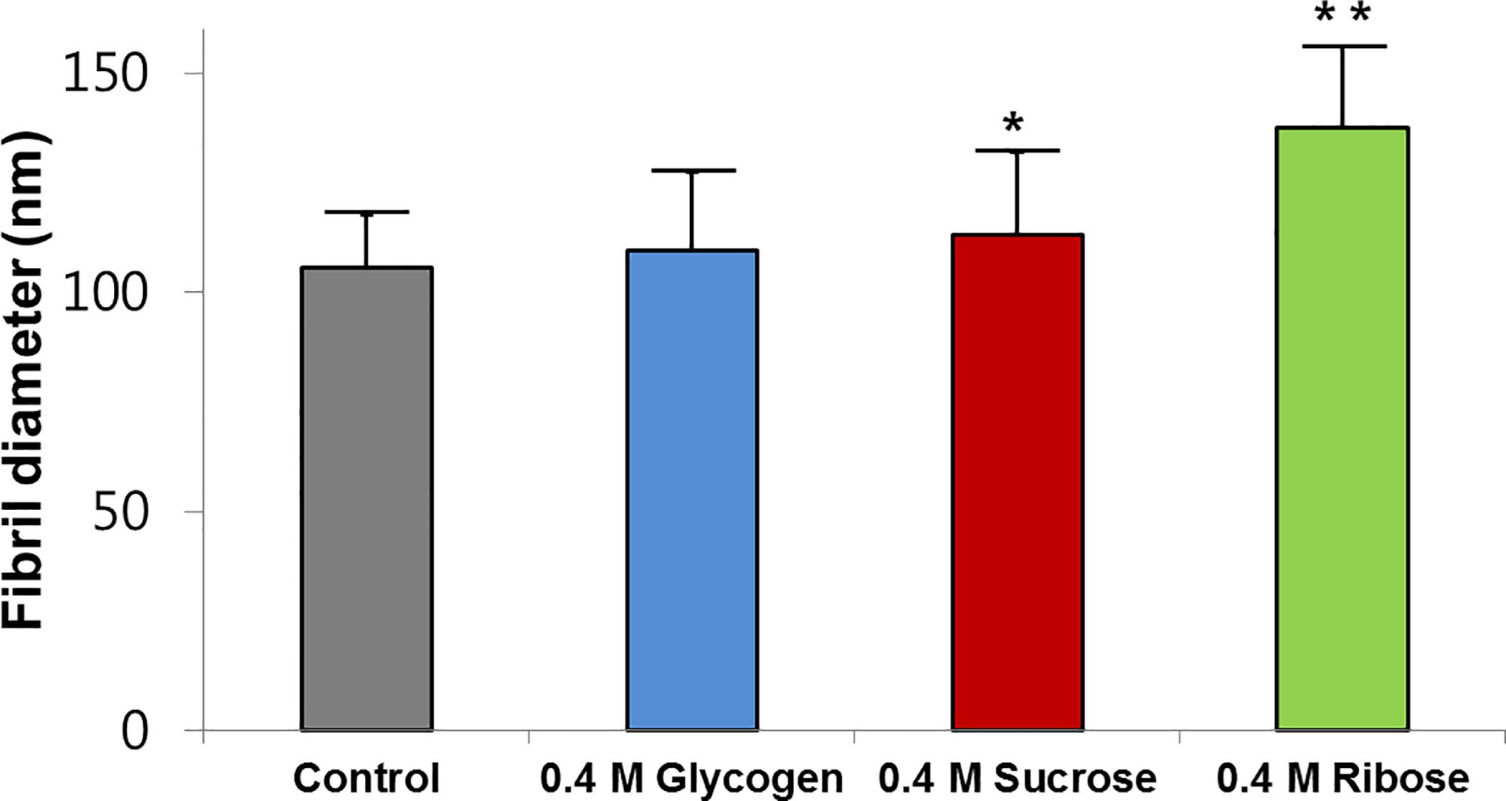
Collagen fibril diameter of rabbit sclera tissue before and after collagen crosslinking using glycation. The fibril diameters were significantly increased following treatment with 0.4 M sucrose or ribose. The 0.4 M ribose group exhibited a significant increase in collagen fibril diameter compared to the 0.4 M sucrose group. *p < 0.05 vs. control scleral tissue; ** p < 0.01 vs. control and 0.4 M sucrose group by Wilcoxon-Mann-Whitney test.

The diameter of normal scleral collagen fibrils was 105.6 ± 21.2 nm and cross-linked scleral collagen fibrils was 109.4 ± 28.8 nm in the 0.4 M glycogen group, 113.1 ± 30.8 nm in the 0.4 M sucrose group, and 137.6 ± 25.3 nm in the 0.4 M ribose group (Fig 5). Collagen fibril diameter were statistically significantly greater in the sucrose and ribose groups compared to the control group (p = 0.004, 0.000, respectively). However, there was no difference in fibril diameter between the glycogen treated and control sclera. In addition, the collagen diameters in the 0.4 M ribose group were thicker than in the 0.4 M sucrose group. (p = 0.000).

### Histological changes

**Fig 6:**
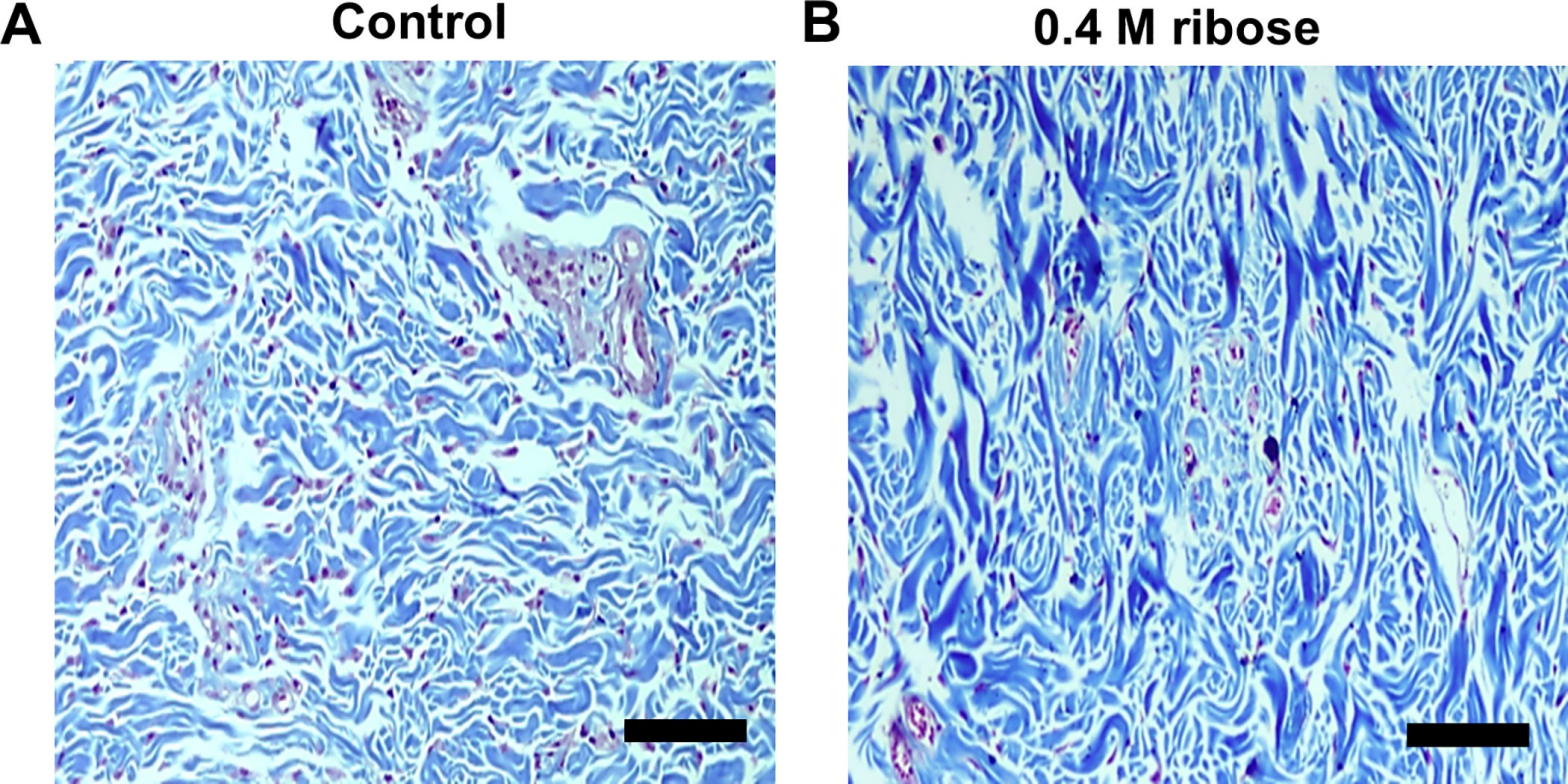
Histology section of normal (A) and 0.4 M ribose-treated (B) rabbit sclera tissues, stained with Masson's trichrome (magnification × 400), where collagen stains blue, nuclei stain black, and cell bodies stain red. The cross-linked scleral tissue showed a dense collagen bundle compared to the normal sclera tissues. Scale bars = 100 μm.

Representative result of the cross-sectional histopathology examination of rabbit scleral tissues before and after collagen crosslinking treatment with 0.4 M ribose and control were shown in Fig 6. The collagen cross-linked scleral tissue showed denser collagen fibril bundles compared to normal scleral tissue. Inflammatory cell infiltration did not differ from normal scleral tissue.

## Discussion

In this study, Raman spectroscopy and AFM were used to evaluate the effect of scleral collagen crosslinking. Raman spectroscopy has been used to analyze the vibrational structure of molecules by measuring the vibrational spectrum of molecules or to qualitatively and quantitatively analyze the materials. Recently, it has been applied to the analysis of biochemical and morphological information of intracellular or extracellular biological tissues. AFM is a tool for investigating the surface nanostructure of tissues and it provides high resolution surface image as well as the three-dimensional ultrastructure of the surface morphology. Our study demonstrated a stiffening effect of crosslinking in in vivo rabbit sclera after sub Tenon injection of three carbohydrates for 12 weeks. In addition, it is the first study to compare the effect of crosslinking between various carbohydrates on a concentration basis.

Pathological myopia is a worldwide and sight-threatening refractive disorder. Until now, various non-surgical methods have been developed to slow the progression of myopia [8–13]. However, since scleral biomechanical weakness and thinning are major contributors to the progression of myopia and, consequently, the axial elongation of the eye, increasing the stiffness of the sclera may be considered a more direct therapeutic modality than non-surgical methods.

Recently, the various scleral collagen crosslinking method has been developed. Collagen, the main structural protein in the extracellular space, provides has several basic functions throughout the various connective tissues in the body. Human scleral tissue contains approximately 50% collagen by weight [27,28]. Collagen consists of a triple helix made of the repetitious amino acid sequence “glycine-X-Y,” where the X and Y positions often correspond to proline and hydroxyl proline, respectively. Hydroxyproline and proline play key roles in collagen stability. Therefore, the Raman shift at 919 cm^-1^(C─C stretching of a proline ring), which indicates molecular changes in collagen as shown in this study. Clinically, collagen crosslinking is performed using UVA and riboflavin mainly in the cornea. Other methods include chemical agents, such as formaldehyde, glyceraldehyde, glutaraldehyde, epoxy compounds, and physical methods, such as dehydrothermal treatment and microwave irradiation [29,30]. However, these methods are limited due to difficulties in accessing the region of interest and the risk of cytotoxicity.

Crosslinking using the glycation method can also increase scleral collagen stiffness. Glycation, also known as the Maillard reaction between reducing sugars and proteins, is the first step in the formation of a more stable collagen complex. During this reaction, the carbonyl group of a sugar reacts reversibly with the free amino group of proteins to form unstable Schiff bases, which then is spontaneously rearranged into a stable Amadori product and Maillard products [31,32]. Choi et al. [26] reported an increase in the stiffness of the sclera using a ribose-based glycation method in an in vitro study of human sclera. Glycation is easy to apply compared to other methods and has a relatively low toxicity. Generally, when analyzing the effect of scleral crosslinking, biomechanical data, including the values of Young's modulus and stress, are measured. However, the present study used a new method that involved Raman spectroscopy and atomic force microscopy to investigate the changes in the chemical bonds and fibril diameter of the collagen fiber. Therefore, it is not possible to directly compare the biomechanical data with our data. Biomechanical data are important in determining the effectiveness of crosslinking; however, microscopic determination of the collagen fibril diameter is also important because previous studies have reported that reduced collagen fibril diameter is associated with myopia [33]. Previously, no study reported a change in the mean scleral collagen fibril diameter following crosslinking in rabbit. Karl et al. measured the collagen fibril diameter after scleral crosslinking mediated by riboflavin and blue light in albino rabbit, but reported the results as a distribution of the thickness of collagen fibrils rather than their mean diameter values [34]. In an in vitro study performed by Choi et al, human scleral collagen fibril diameter increased by 22% relative to that of control after riboflavin-UVA crosslinking at 370 nm [16]. Similarly, Wang and Corpuz reported that the collagen fibril diameter increased from 102.56 ± 9.82 nm to 123.54 ± 17.55 nm after genipin-induced scleral crosslinking in a guinea pig model, which corresponds to approximately 20% increase [35]. In our study, the collagen fibril diameter increased by 30% (from 105.6 ± 21.2 nm to 137.6 ± 25.3 nm) when 0.4 M ribose was used. Therefore, the effect of 0.4 M ribose-induced crosslinking on the collagen fibril diameter is quantitatively superior. However, this observation should be interpreted with caution because stiffness of sclera and effects of crosslinking may differ between humans and animals. According to Zhang et al, human sclera is three times stiffer than rabbit sclera, and riboflavin-UVA crosslinking increases the biomechanical stiffness of rabbit sclera but not human sclera [36]. Therefore, biomechanical testing including Young's modulus may be necessary in future studies.

In this study, collagen crosslinking using carbohydrates was found to produce stronger tissue when the monosaccharide ribose, rather than disaccharides and polysaccharides, was used. During the non-enzymatic glycation of proteins, the carbonyl group of a sugar reacts with the amino group of a protein. A reducing sugar has a free carbonyl group and all monosaccharides are reducing sugars. Sucrose and glycogen, however, are a non-reducing carbohydrate and must be hydrolyzed before it can proceed through the collagen crosslinking reaction. It is known that glycation is proportional to the percentage of the open-chain form and inversely proportional to the carbon chain length of the sugar [37]. Therefore, it can be presumed that ribose, which is a monosaccharide, is predominantly present in the open-chain form at the same dose, and this may have contributed to our observation in this study that ribose-mediated crosslinking resulted in the strongest bonds. For crosslinking to be effective, the sugar molecule must penetrate the sclera. The monosaccharide ribose has a molecular weight of 150 Da, and thus it is smaller than sucrose (342 Da) and glycogen (666 Da). Consequently, a larger amount of ribose presumably penetrated the sclera and participated in collagen crosslinking.

The strength of scleral crosslinking was greater for carbohydrates at higher concentrations. This is likely because there are more carbonyl groups that can participate in crosslinking. In sucrose-treated rabbits, the strength of scleral crosslinking was stronger than that of control. It is known that the hydrolysis of sucrose and glycogen occurs slowly under natural conditions, with both sugars being able to remain relatively stables over years. Generally, sugar's hydrolysis is known to accelerate in a hot and acidic environment. Therefore, it is possible that hydrolysis was accelerated by the hot and acidic environment of the body.

In this study, ribose was used as a monosaccharide. In fact, glucose plays an important role in hyperglycemia and diabetes in the natural glycation process. Thus, using glucose is more likely to mimic the in vivo process of glycation. Nevertheless, the reason for using ribose instead of glucose in this study is that pentoses have higher reactivity of crosslinking than hexoses. Previous studies comparing glucose and ribose crosslinking in rat skin and tendon in vitro showed a quicker increase in crosslinking in ribose treated tissue [38].

In the AFM results, the collagen fibril diameter was significantly increased in the sclera that was treated with sucrose and ribose compared to the control (Fig 4). Interestingly, the morphological changes were more pronounced in the ribose group than in the sucrose group, which was consistent with Raman spectra results in this study. These results suggest that higher concentrations of ribose are more effective at achieving scleral crosslinking using glycation.

During histologic analysis, the collagen density was found to be increased in the ribose-treated group compared to the control group. Moreover, despite the use of an elevated concentration of ribose at 0.4 M, we observed no inflammatory reaction in the tissue, which indicates that there is less tissue toxicity. Additionally, the histologic findings from this study suggest that collagen crosslinking treatment through glycation is an effective and safe treatment against progressive myopic sclera.

A limitation of the study was the lack of a direct comparison of the strength of collagen crosslinking between conventional crosslinking by UVA and riboflavin and the carbohydrate methods. Second limitation is that Raman Spectroscopy, which does not directly measure the stiffness of sclera. In the future study, mechanical tests should be added to the experiments. Third limitation is that we did not evaluate the retinal and conjunctival toxicity by concentration through histological analysis. Subconjunctival fibrosis is a problem in the ophthalmic area because of the difficulty in surgical operation during filtering surgery such as trabeculectomy in glaucoma surgery. Therefore, in future studies, it is necessary to evaluate the retinal and conjunctival toxicity and safety of crosslinking using glycation. Finally, the sclera tissue used for the analysis is the sclera tissue of the subTenon injection site, not the posterior sclera, which directly affects the axial elongation of the eye ball. Despite its limitation, we confirmed that glycation crosslinking can be used as a treatment option for myopic progression and that a monosaccharide exhibited stronger crosslinking compared to a disaccharide or polysaccharide. Ultimately, this technique may represent a potential therapeutic avenue for delaying the progression of myopia, but more research is needed to optimize the application of treatment as well as to evaluate its biochemical effects on the retina and surrounding ocular tissues.

In conclusion, scleral crosslinking with carbohydrates can enhance the strength of the collagen bond compared to normal sclera. When compared at the same concentration, the monosaccharide ribose produced stronger crosslinking than did sucrose and glycogen. These in vivo results suggest that glycation may be a new approach to a scleral-based treatment for inhibiting myopia progression.

## Supporting information

S1 Data Supplement DATA(JIN). (XLSX)
